# Improved Photoelectrochemical Performance of MoS_2_ through Morphology-Controlled Chemical Vapor Deposition Growth on Graphene

**DOI:** 10.3390/nano11061585

**Published:** 2021-06-17

**Authors:** Dong-Bum Seo, Tran Nam Trung, Sung-Su Bae, Eui-Tae Kim

**Affiliations:** Department of Materials Science & Engineering, Chungnam National University, Daejeon 34134, Korea; sdb987@naver.com (D.-B.S.); trannamtrung@qnu.edu.vn (T.N.T.); bss1007@naver.com (S.-S.B.)

**Keywords:** photoelectrocatalysis, 2D nanostructures, MoS_2_, graphene, heterojunction

## Abstract

The morphology of MoS_2_ nanostructures was manipulated from thin films to vertically aligned few-layer nanosheets on graphene, in a controllable and practical manner, using metalorganic chemical vapor deposition. The effects of graphene layer and MoS_2_ morphology on photoelectrochemical (PEC) performance were systematically studied on the basis of electronic structure and transitions, carrier dynamic behavior, and PEC measurements. The heterojunction quality of the graphene/vertical few-layer MoS_2_ nanosheets was ensured by low-temperature growth at 250−300 °C, resulting in significantly improved charge transfer properties. As a result, the PEC photocurrent density and photoconversion efficiency of the few-layer MoS_2_ nanosheets significantly increased upon the insertion of a graphene layer. Among the graphene/MoS_2_ samples, the few-layer MoS_2_ nanosheet samples exhibited shorter carrier lifetimes and smaller charge transfer resistances than the thin film samples, suggesting that vertically aligned nanosheets provide highly conductive edges as an efficient pathway for photo-generated carriers and have better electronic contact with graphene. In addition, the height of vertical MoS_2_ nanosheets on graphene should be controlled within the carrier diffusion length (~200 nm) to achieve the optimal PEC performance. These results can be utilized effectively to exploit the full potential of two-dimensional MoS_2_ for various PEC applications.

## 1. Introduction

The two-dimensional (2D) transition metal dichalcogenides, such as MoS_2_ and WSe_2_, have recently emerged as promising photocatalysts of photoelectrochemical (PEC) water-splitting applications because of their excellent catalytic activity, high chemical stability, and earth abundance [1,2]. Two-dimensional MoS_2_ is of particular interest because of its high carrier mobility (a few hundred cm^2^/V·s), high optical absorption, and eco-friendliness. The PEC efficiency of MoS_2_ is significantly affected by the design and implementation of an appropriate heterostructure that can enhance the separation and subsequent transfer of photogenerated electron–hole (e–h) pairs using a built-in potential generated by the heterojunction [3,4,5]. Among various heterostructures, such as MoS_2_/TiO_2_, MoS_2_/ZnO, and MoS_2_/CdS [5,6,7,8,9], 2D MoS_2_ nanostructures/graphene has attracted considerable attention as promising PEC cathode and anode material systems [10,11]. Graphene is considered the most fascinating conducting layer because it exhibits remarkable electron mobility (>15,000 cm^2^·V^−1^·s^−1^) [12] and forms a favorable heterojunction with MoS_2_ for efficient charge separation and transfer [13]. Chang et al. reported that graphene in MoS_2_/graphene–CdS composites improves the charge transfer ability and retards the recombination of e–h pairs, thereby enhancing photocatalytic hydrogen evolution reactions [13]. Yu et al. demonstrated a one-pot synthesis of CdS/MoS_2_/graphene hollow spheres for highly efficient photocatalytic hydrogen evolution reaction [14]. Carraro et al. showed that the p–n heterojunction of MoS_2_/crumpled graphene enhances PEC hydrogen production [11]. Zhang et al. reported that the built-in electric field of 2D MoS_2_/reduced graphene oxide heterojunctions suppresses the recombination of e–h pairs and promotes PEC efficiency [15]. However, most MoS_2_/graphene composites exhibit randomly assembled structures of 2D MoS_2_ and graphene. The heterostructure of few-layer MoS_2_ nanosheets aligned vertically on graphene substrates has not been implemented by wet-chemical approaches.

The architecture configuration of few-layer MoS_2_ on electrode substrates is another important factor that can be leveraged to improve PEC efficiency because of its 2D layered structure comprised of a strong in-plane covalent bonding of S–Mo–S and a weak out-of-plane van der Waals interaction between neighboring S–S layers. The vertically standing 2D MoS_2_ structure has recently been reported to enhance PEC performance considerably, because the highly conductive edges of 2D MoS_2_ provide an efficient pathway for photo-excited carriers and have good electronic contact with the substrates [8,16,17]. He et al. demonstrated that the edge-on structure of MoS_2_ flakes/TiO_2_ nanowires improves the photocatalytic hydrogen evolution of MoS_2_ [16]. Recently, we reported the enhanced PEC water-splitting activity of few-layer MoS_2_ nanosheets vertically grown on indium-tin oxide (ITO) and TiO_2_ nanowires [17,18]. In addition, the morphology of MoS_2_ affected the adhesion behavior of as-formed gas bubbles. Lu et al. reported that a superaerophobic surface of vertically stacked MoS_2_ flake electrodes significantly improved PEC hydrogen evolution reactions [19].

**Figure 1:**
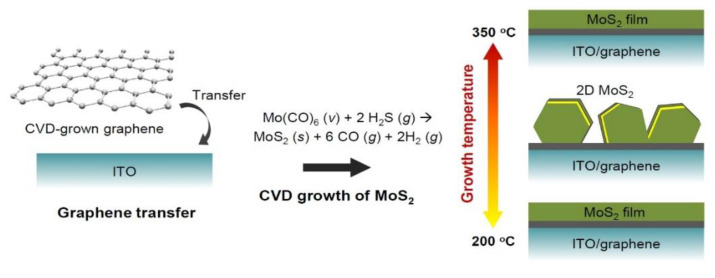
Schematic of the preparation method for various MoS_2_ nanostructures on graphene.

The thickness controllability of 2D MoS_2_ nanosheets is also an important factor to maximize its PEC activity, because the bandgap energy is tunable from ~1.2 eV for the indirect gap of the bulk form to ~1.9 eV for the direct gap of the monolayer [20,21,22]. The theoretical limiting efficiency of single-junction cells can be achieved at a bandgap energy of 1.59 eV, which corresponds to that of few-layer MoS_2_. Velicky et al. revealed that few-layer (5–10) MoS_2_ flakes provide a good compromise between large surface areas and sufficiently fast charge-carrier transport for energy storage and energy conversion applications [23]. However, information on morphology-controlled synthesis, including the thickness, size, and architecture of 2D MoS_2_ on graphene and its systematic electronic, optical, and PEC properties, is minimal. Herein, we report the controllable growth of few-layer MoS_2_ nanosheets on graphene by using metalorganic chemical vapor deposition (MOCVD) for PEC water-splitting applications. The morphology of MoS_2_ was successfully manipulated from thin film to vertically aligned few-layer nanosheets on graphene via a controllable and practical manner, that is, by varying growth temperature (Figure 1). Furthermore, MoS_2_ was grown on graphene at relatively low temperatures (≤ 350 °C) in order to minimize the structural and chemical destruction of the graphene layer. The beneficial effects of the graphene layer were also systematically studied. For PEC water-splitting applications, the optimal structure of MoS_2_ on graphene, including PEC properties, electronic structures and carrier transfer properties across the graphene/MoS_2_ heterojunction, was determined by a systematic study.

## 2. Experimental

The Graphene was grown on Cu foils (Alfa Aesar) using inductively-coupled plasma chemical vapor deposition (ICP CVD) with CH_4_ and H_2_ gases at 950 °C for 5 min. The ICP power and growth pressure were fixed at 200 W and 1 Torr, respectively. The synthesized graphene on Cu was transferred on an ITO glass substrate (Figure 1). The CVD growth and transfer procedures of graphene were described in further detail elsewhere [24]. MoS_2_ was grown on ITO and ITO/graphene substrates at various temperatures (200 °C, 250 °C, 300 °C, and 350 °C) through MOCVD reaction with Mo(CO)_6_ and H_2_S gas (5 vol% in balance N_2_) as Mo and S precursors, respectively. Mo(CO)_6_ was vaporized at 20 °C and carried into a quartz reaction tube with Ar gas of 25 standard cubic centimeters per minute (SCCM). The flow rate of H_2_S gas was 75 SCCM. The growth pressure and time were fixed at 1 Torr and 5 min, respectively.

The morphology and microstructure of MoS_2_ were characterized via scanning electron microscopy (SEM: Hitachi S-4800, Tokyo, Japan) and transmission electron microscopy (TEM: Tecnai G^2^ F30 S-Twin, Hillsboro, OR, USA). The crystal structure of MoS_2_ was characterized by TEM and micro-Raman spectroscopy using an excitation band of 532 nm and a charge-coupled device detector. The optical properties were evaluated by ultraviolet–visible (UV–Vis) spectroscopy (S-3100, SCINCO, Seoul, Korea) and photoluminescence (PL) spectroscopy (excitation at 532 nm). The photo-excited carrier behavior was investigated by time-resolved PL (TRPL) measurements. The samples were excited using a 467 nm pulsed laser and the transient signal was recorded using a time-correlated single photon counting spectrometer (Horiba Fluorolog 3, Kyoto, Japan). The energy level of MoS_2_ was evaluated via UV photoelectron spectroscopy (UPS; Thermo scientific, K-alpha^+^, Waltham, MA, USA).

PEC cells were fabricated on 1 × 2 cm^2^ ITO and ITO/graphene substrates. The working area of the PEC cells was fixed at 0.5 × 0.5 cm^2^ using nonconductive epoxy to cover undesired areas. PEC characterization was performed using a three-electrode system and an electrochemical analyzer (potentiostat/galvanostat 263A, HS Technologies, Gyeonggi-do, Korea). A Pt plate and KCl-saturated calomel (Hg/Hg_2_Cl_2_) were used as counter and reference electrodes, respectively. The electrolyte solution was prepared with 0.3 M KH_2_PO_4_ in KOH solution (pH 6.5). The light source used was a 150 W Xe arc lamp that delivered an intensity of 100 mW/cm^2^ of simulated AM 1.5 G irradiation. The current density–voltage characteristics were recorded using a source meter (Keithley 2400, Cleveland, OH, USA). Electrochemical impedance spectroscopy (EIS) measurement was performed under constant light illumination (100 mW/cm^2^) at a bias of 0.6 V while varying the AC frequency from 100 kHz to 100 mHz.

## 3. Results and Discussion

**Figure 2:**
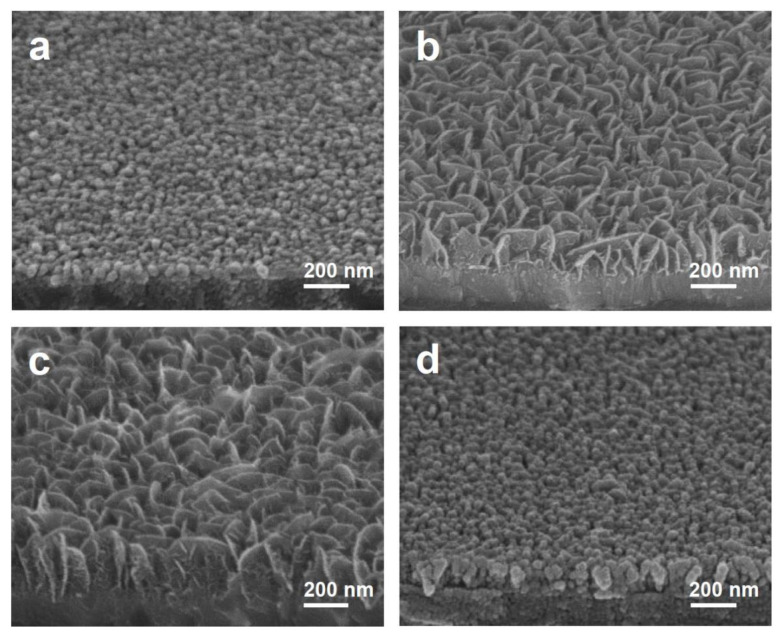
SEM images of MoS_2_ nanostructures synthesized on graphene at various growth temperatures: (**a**) G/MoS_2_-200 at 200 °C, (**b**) G/MoS_2_-250 at 250 °C, (**c**) G/MoS_2_-300 at 300 °C, and (**d**) G/MoS_2_-350 at 350 °C.

The morphology of MoS_2_ grown on graphene was crucially affected by the growth temperature of the MOCVD process. At 200 °C, an MoS_2_ film with a thickness of ~50 nm was formed, hereinafter referred to as G/MoS_2_-200 (Figure 2a). When the growth temperature was increased to 250 °C, the MoS_2_ morphology drastically changed to vertically aligned nanosheets with a height of ~200 nm and length of ~150–250 nm, hereinafter referred to as G/MoS_2_-250 (Figure 2b). The MoS_2_ nanosheets were vertically aligned and densely packed on the ITO/graphene substrate, which is ideal for PEC photoelectrode applications due to its high specific surface area of 2D MoS_2_ catalytic edge sites. Theoretical and experimental results indicate that the strong catalytic activity of 2D MoS_2_ arises from active S atom sites exposed along the edges [3,4,5]. The height of the vertically aligned MoS_2_ nanosheets increased further to ~250 nm at 300 °C, hereinafter referred to as G/MoS_2_-300 (Figure 2c). However, above 350 °C, the morphology of MoS_2_ changed back to a ~130 nm-thick film, hereinafter referred to as G/MoS_2_-350 (Figure 2d). The size of 2D layered MoS_2_ nanosheets (S–Mo–S) seemed to be determined by the migration length of impinged Mo adatoms, which bonds two S anions in S-stabilized growth condition. The height of the G/MoS_2_-300 nanosheet was higher than that of G/MoS_2_-250 because of the enhanced migration length of Mo adatoms at a higher growth temperature. When the growth temperature was increased further, the ratio of Mo to S adatoms also increased due to the predominant desorption of S adatoms from the growth front surface. Under S-deficient conditions, impinged Mo adatoms tended to bond immediately with the nearest Mo adatoms. As a result, a thin film morphology composed of particles was observed at 350 °C [8,25,26].

**Figure 3:**
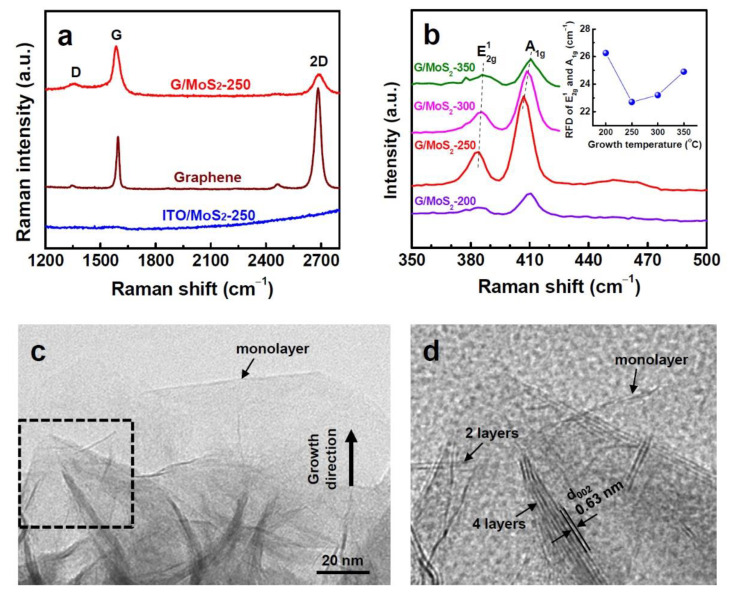
(**a**) Raman spectra of ITO/MoS_2_-250, G/MoS_2_-250, and pristine graphene. (**b**) Raman spectra of G/MoS_2_-200, G/MoS_2_-250, G/MoS_2_-300, and G/MoS_2_-350. The inset shows the RFD between the E^1^_2g_ and A_1g_ Raman modes. (**c**,**d**) low-magnification and high-resolution TEM images of MoS_2_ nanosheets of G/MoS_2_-250, respectively.

The structures of MoS_2_ and graphene were investigated using Raman spectroscopy and TEM. The Raman spectrum of pristine CVD-grown graphene showed a low-intensity ratio of D to G band peaks (>0.15) (Figure 3a). The light transmittance of 96.8 % at 550 nm (Figure S1 in Supporting Information) corresponds to approximately one and a half layers of high-quality graphene [24]. The graphene layer was still present after the MOCVD growth of MoS_2_, as confirmed by the presence of characteristic G and 2D band peaks in the Raman spectrum (Figure 3a). All the graphene/MoS_2_ samples exhibited the E^1^_2g_ mode and A_1g_ mode in Raman spectra (Figure 3b). The E^1^_2g_ and A_1g_ modes are attributed to the in-plane vibration of Mo and S atoms and the out-of-plane vibration of S atoms, respectively. The number of MoS_2_ layers can be estimated from the positions and relative frequency difference (RFD) of the E^1^_2g_ and A_1g_ peaks [27,28]. For G/MoS_2_-250, the RFD value (22.3 cm^−1^) of E^1^_2g_ (385.0 cm^−1^) and A_1g_ peaks (407.3 cm^−1^) corresponds to a few layers of MoS_2_. The increased RFD value (24.0 cm^−1^) with a blue-shifted A_1g_ peak (409.3 cm^−1^) of G/MoS_2_-300 indicates a slightly increased number of layers. The blue-shifted A_1g_ mode is attributed to the increased restoring force of the interlayer S–S atoms due to the increased number of layers. The thin film samples (G/MoS_2_-200 and G/MoS_2_-350) exhibited further increased RFD values with more-blue-shifted A_1g_ modes (inset in Figure 3b). The TEM images of G/MoS_2_-250 showed that the MoS_2_ nanosheets have a layered structure (Figure 2c). The nanosheets comprised several (1–5) layers with an interlayer spacing of 0.63 nm, corresponding to semiconducting 2H MoS_2_ (Figure 2d). Meanwhile, the MoS_2_ nanosheets grown on ITO at 250 °C, hereinafter referred to as ITO/MoS_2_-250, exhibited similar size and morphology as the counterpart sample, G/MoS_2_-250 (Figure S2a). The RFD value of the E^1^_2g_ and A_1g_ peaks of ITO/MoS_2_-250 was also nearly the same as that of G/MoS_2_-250 (Figure S2b).

**Figure 4:**
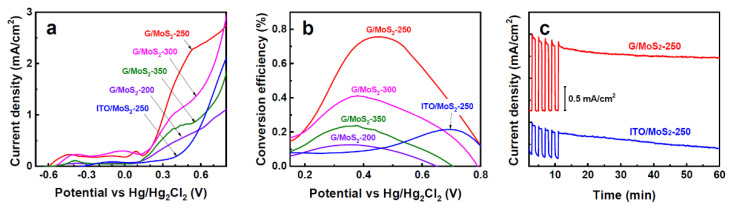
(**a**) Photocurrent density–potential curves and (**b**) photoconversion efficiency–potential curves of PEC cells with various working electrodes (ITO/MoS_2_-250, G/MoS_2_-200, G/MoS_2_-250, G/MoS_2_-300, and G/MoS_2_-350). (**c**) Photocurrent–time plots for ITO/MoS_2_-250 and G/MoS_2_-250 at 0.6 V.

The PEC activities of the MoS_2_ samples were evaluated by recording linear sweep voltammograms in the dark and under simulated AM 1.5 G illumination. Compared with ITO/MoS_2_-250, G/MoS_2_-250 showed significantly higher PEC photocurrent density through the measured potential range (Figure 4a), whereas the dark currents of the two samples were comparable to each other (Figure S3). Therefore, G/MoS_2_-250 yielded approximately three times higher photoconversion efficiency (0.76% at 0.45 V) than ITO/MoS_2_-250 (0.22% at 0.7 V), as shown in Figure 4b. The photoconversion efficiency of G/MoS_2_-250 was comparable with various recently reported photoanodes, such as TiO_2_/MoS_2_ [9,29,30], ZnO/MoS_2_ [31], CoTe/MoS_2_ [32], and MoS_2_/α-Fe_2_O_3_ [33]. Moreover, the long-term stability of MoS_2_ flakes was significantly improved by forming a heterojunction with graphene (Figure 4c). The photocurrent of G/MoS_2_-250 did not change significantly through 1 h of illumination, whereas the photocurrent of ITO/MoS_2_-250 decreased continuously. The decayed photocurrent of ITO/MoS_2_-250 can be attributed to the decomposition of MoS_2_, mainly the loss of S elements [8]. The improved stability can be attributed to the effective separation and transfer of the photogenerated e–h pairs in the heterojunction [8]. Among the graphene/MoS_2_ samples, the vertically aligned MoS_2_ nanosheet samples (G/MoS_2_-250 and G/MoS_2_-300) yielded significantly higher photocurrent densities compared with the MoS_2_ thin film samples (G/MoS_2_-200 and G/MoS_2_-350). PEC activity was also significantly affected by the size of the few-layer MoS_2_ nanosheet. Despite its larger MoS_2_ nanosheet size, G/MoS_2_-300 yielded a lower photocurrent density than G/MoS_2_-250. Our previous study on MoS_2_ nanosheets on TiO_2_ nanowires demonstrated that for PEC applications, the optimum size of MoS_2_ nanosheets seemed to depend on the diffusion length of the carriers (~0.34 and ~0.24 μm for electrons and holes, respectively) [18].

**Figure 5:**
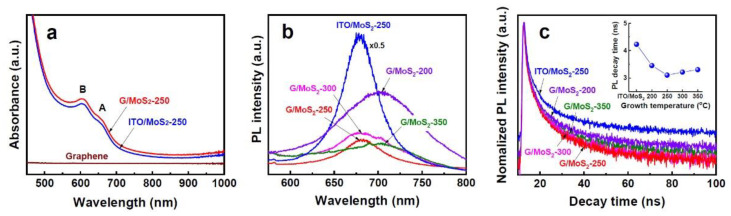
(**a**) UV–Vis absorption spectra of ITO/MoS_2_-250, G/MoS_2_-250, and pristine graphene. (**b**) PL spectra and (**c**) TRPL results of ITO/MoS_2_-250, G/MoS_2_-200, G/MoS_2_-250, G/MoS_2_-300, and G/MoS_2_-350. The inset in (**c**) shows the carrier lifetimes extracted from corresponding TRPL measurements.

To investigate the electronic transitions and charge transport properties of MoS_2_ nanosheet samples, systematic studies, including EIS, UV–Vis absorption, PL, and TRPL spectroscopy, were conducted. G/MoS_2_-250 and ITO/MoS_2_-250 showed nearly the same UV–Vis absorption spectra with two prominent peaks at approximately 607 nm and 663 nm (Figure 5a). The two peaks, known as excitons B and A, respectively, can be attributed to the direct excitonic transitions at the *K* point of the MoS_2_ Brillouin zone [8,34]. The absorption difference of the two samples resulted from only the optical absorption of the graphene layer (<5%).

Both samples exhibited a PL peak at 676 nm (Figure 5b), which is consistent with the energy of exciton A, suggesting that a dominant electronic transition was the direct bandgap transitions at the *K* point. G/MoS_2_-300 showed a main PL peak at 676 nm, with a satellite peak at 705 nm. The thin film samples (G/MoS_2_-200 and G/MoS_2_-350) yielded a red-shifted PL peak at 705 nm with respect to G/MoS_2_-250. The PL around 705 nm is attributed to the indirect bandgap transitions of the MoS_2_ bulk form. All graphene/MoS_2_ samples achieved significantly lower PL efficiencies than ITO/MoS_2_-250. The considerable PL quenching can be attributed to the reduced recombination of e–h pairs through the heterojunction of graphene/MoS_2_. The dynamic behavior of photo-generated carriers was further investigated by TRPL spectroscopy (Figure 5c). The average carrier lifetimes were extracted by the PL decay kinetics fitted by a bi-exponential decay profile [35]. G/MoS_2_-250 exhibited the shortest carrier lifetime of 3.09 ns, whereas ITO/MoS_2_-250 showed the longest carrier lifetime of 4.23 ns (inset in Figure 5c). The reduced carrier lifetime indicates that the heterojunction of graphene/MoS_2_ is beneficial to the efficient separation and transport of photo-generated carriers to the semiconductor/liquid interface [14]. In addition, the few-layer MoS_2_ nanosheet samples (G/MoS_2_-250 and G/MoS_2_-300) exhibited shorter carrier lifetimes than the thin film samples (G/MoS_2_-200 and G/MoS_2_-350), suggesting that vertically aligned nanosheets provided more efficient carrier transport paths, i.e., they have highly conductive edges compared with the bulk form.

**Figure 6:**
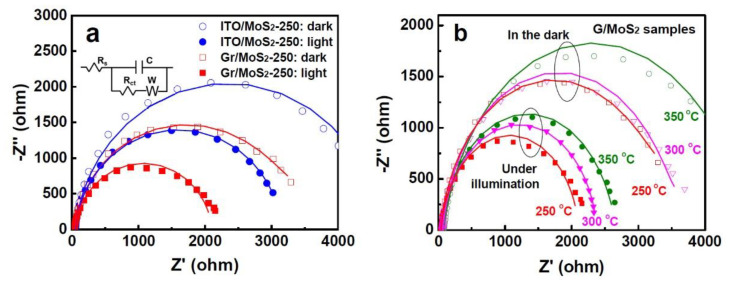
(**a**) Nyquist plots of ITO/MoS_2_-250 and G/MoS_2_-250 in the dark and under illumination. The inset shows an equivalent Randles circuit. (**b**) Nyquist plots of G/MoS_2_-250, G/MoS_2_-300, and G/MoS_2_-350.

Figure 6a shows the Nyquist plots of the EIS spectra of G/MoS_2_-250 and ITO/MoS_2_-250 in the dark and under illumination. G/MoS_2_-250 showed smaller EIS semicircles than ITO/MoS_2_-250, whose radius mirrors the charge transfer resistance (*R_ct_*), indicating that the graphene layer significantly enhanced the charge transfer efficiency. The Nyquist plots can be fitted using a simplified Randles circuit (inset in Figure 6a), which consists of *R_ct_*, solution resistance (*R_s_*), constant phase element (*Q*), and diffusion of species in electrolyte solution represented by Warburg impedance (*W*). G/MoS_2_-250 yielded values of 3196 Ω and 2069 Ω for *R_ct_* in the dark and under illumination, respectively, whereas ITO/MoS_2_-250 exhibited *R_ct_* values of 4597 Ω and 3166 Ω. Moreover, the *R_ct_* (dark) to *R_ct_* (photo) ratio (1.55) of G/MoS_2_-250 was greater than that of ITO/MoS_2_-250 (1.45), suggesting that graphene/MoS_2_ heterojunction was more beneficial under illumination than in the dark. G/MoS_2_-250 and G/MoS_2_-300 showed smaller EIS semicircles than G/MoS_2_-350, indicating a decrease in *R_ct_* and suppression of charge recombination (Figure 6b). The improved PEC activity and lower *R_ct_* can be attributed to the desirable vertically aligned architecture, which provided highly conductive edges as an efficient pathway for photo-generated carriers and better electronic contact with graphene substrates [16].

**Figure 7:**
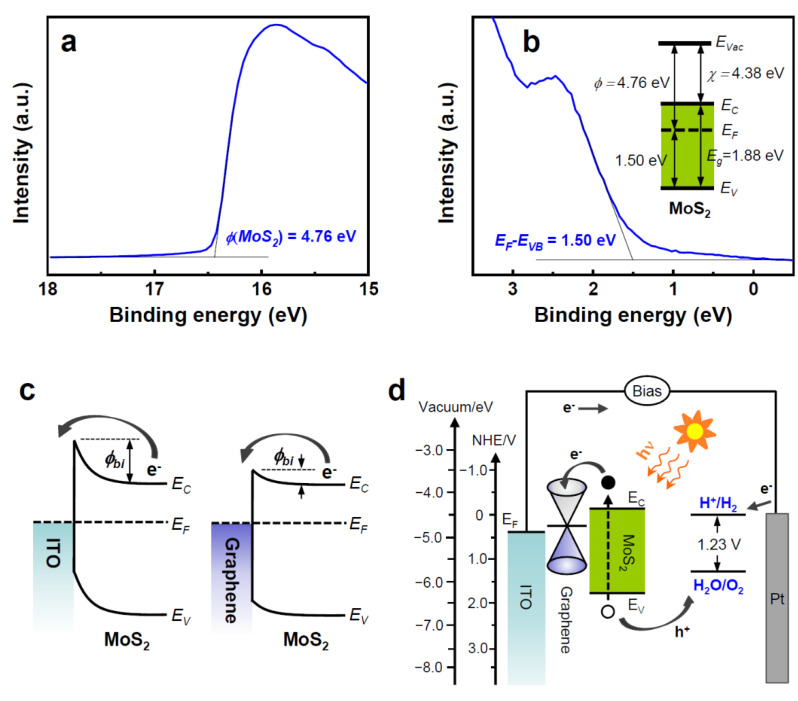
(**a**) UPS secondary electron cut-off and (**b**) valence spectra of G/MoS_2_-250. The inset in (**b**) shows the corresponding energy band diagram of 2D MoS_2_ nanosheets on graphene. (**c**) Energy band diagrams of the Schottky junctions of ITO/MoS_2_ and graphene/MoS_2_. (**d**) PEC water-splitting working principle of 2D MoS_2_ nanosheets on graphene.

To understand the carrier transport property across the heterojunction of graphene/MoS_2_, its electronic structure was studied via UPS. Figure 7a,b shows the UPS secondary electron cut-off and valence spectra of G/MoS_2_-250, respectively. The work function of MoS_2_ was 4.76 ± 0.15 eV, which can be determined by the difference between the photon energy of excited radiation (21.2 eV) and the spectrum width, which is measured from the valence band and secondary edges (16.44 eV, Figure 7a). The energy difference between the Fermi level and valence band edge (*E_F_*−*E_VB_*) was 1.50 eV (Figure 7b). Considering the bandgap energy of ~1.88 eV for MoS_2_ determined by the UV–Vis absorption and PL spectra, the electron affinity (*χ*) of MoS_2_ was approximately 4.38 eV, which is consistent with previously reported values (~4.3 eV) [36]. The estimated electronic structure of MoS_2_ nanosheets is shown in the inset of Figure 7b. The Fermi level of MoS_2_ is close to the Fermi level (~4.6–4.8 eV of work function) of pristine few-layer graphene [37], resulting in a small built-in potential barrier for electron transport (Figure 7c). Consequently, G/MoS_2_-250 exhibited a small positive water oxidation onset potential (~0.18 V, as shown in Figure 4a), which is generally defined by the potential at the intersection of the dark current and the tangent at the maximum slope of the photocurrent. By contrast, ITO/MoS_2_-250 showed a water oxidation onset potential of ~0.49 V, implying a larger built-in potential barrier for electron transport (Figure 7c). The appropriately located Fermi level of graphene between the Fermi level of ITO and the conduction band edge of MoS_2_ is another benefit of the graphene/MoS_2_ heterostructure for the efficient extraction of electrons to the cathode (Figure 7d).

## 4. Conclusions

We demonstrated the successful manipulation of MoS_2_ morphology from thin film to vertically-aligned few-layer nanosheets on graphene in a controllable and practical manner using MOCVD. Desirable vertical few-layer MoS_2_ nanosheets were synthesized on graphene at relatively low temperatures (250–300 °C). Low-temperature growth was beneficial to the formation of high-quality graphene/MoS_2_ heterojunctions, which not only significantly enhanced the charge transfer resistance but also exhibited cathodic-shifted water oxidation onset potential (~0.18 V) by lowering a built-in potential barrier for electron transport. As a result, G/MoS_2_-250 showed approximately three times higher photoconversion efficiency (0.76% at 0.45 V) than ITO/MoS_2_-250 (0.22% at 0.7 V). The best PEC performance of G/MoS_2_-250 resulted from the combined effect of (i) a favorable graphene/MoS_2_ heterojunction, through which photo-generated e–h pairs were efficiently separated and transported; (ii) a desirable architecture of vertically aligned few-layer MoS_2_ nanosheets, which provided highly conductive edges that serve as efficient carrier pathways and better electronic contact with the graphene substrate; and (iii) a controlled height of few-layer MoS_2_ nanosheets within the diffusion length of the carriers. These results not only provide the optimal morphology of MoS_2_ for exploiting the full potential of 2D MoS_2_ for various PEC applications, but also demonstrate a practical large-scale and controllable 2D MoS_2_ synthesis approach on graphene.

## Data Availability

Data are available in the main text.

## Supplementary Materials

The following are available online at https://www.mdpi.com/article/10.3390/nano11061585/s1, Figure S1: UV–Vis absorption spectrum of pristine graphene, exhibiting a light transmittance of 96.88 % at 550 nm, Figure S2: (a) SEM image of few-layer MoS2 nanosheets grown on ITO (ITO/MoS2-250). (b) Raman spectra of ITO/MoS2-250, G/MoS2-250, and pristine graphene, Figure S3: Dark current density–potential curves of PEC cells with various working electrodes (ITO/MoS2-250, G/MoS2-200, G/MoS2-250, G/MoS2-300, and G/MoS2-350).
