# Supplementary material for: Improved Photoelectrochemical Performance of MoS2 through Morphology-Controlled Chemical Vapor Deposition Growth on Graphene
Source: Nanomaterials (Basel). 2021 Jun 17;11(6):1585. doi: 10.3390/nano11061585 (PMC8235607; doi:10.3390/nano11061585)
Supplement: Supplementary file 1 [file nanomaterials-11-01585-s001.zip › nanomaterials-1263816-supplementary.pdf]

nanomaterials

*Supporting Information*

# Improved Photoelectrochemical Performance of $MoS_2$ through Morphology-Controlled Chemical Vapor Deposition Growth on Graphene

**Dong-Bum Seo, Tran Nam Trung, Sung-Su Bae and Eui-Tae Kim ***

Department of Materials Science & Engineering, Chungnam National University, Daejeon 34134, Korea; sdb987@naver.com (D.-B.S.); trannamtrung@qnu.edu.vn (T.N.T); bss1007@naver.com (S.-S.B.)
* Correspondence: etkim@cnu.ac.kr

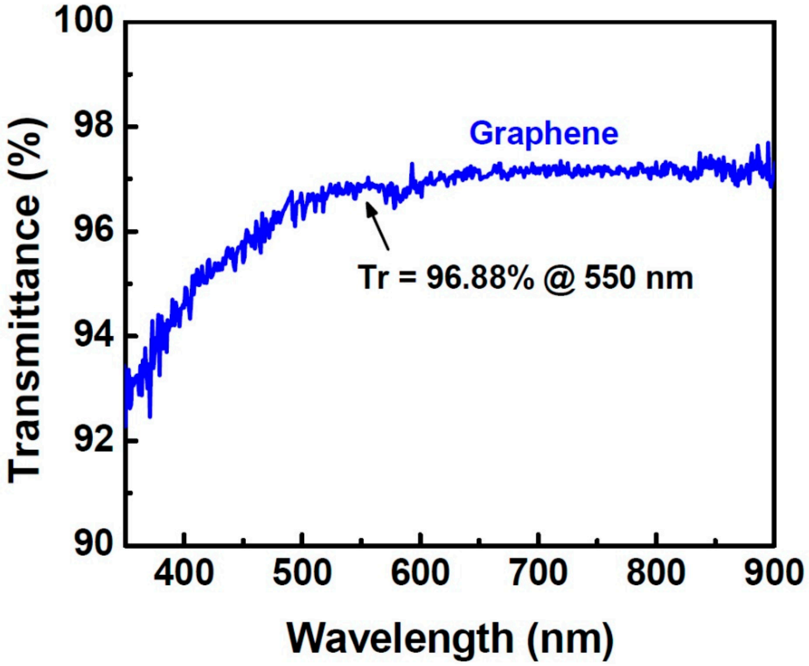


**Figure S1.** UV–Vis absorption spectrum of pristine graphene, exhibiting a light transmittance of 96.88 % at 550 nm.

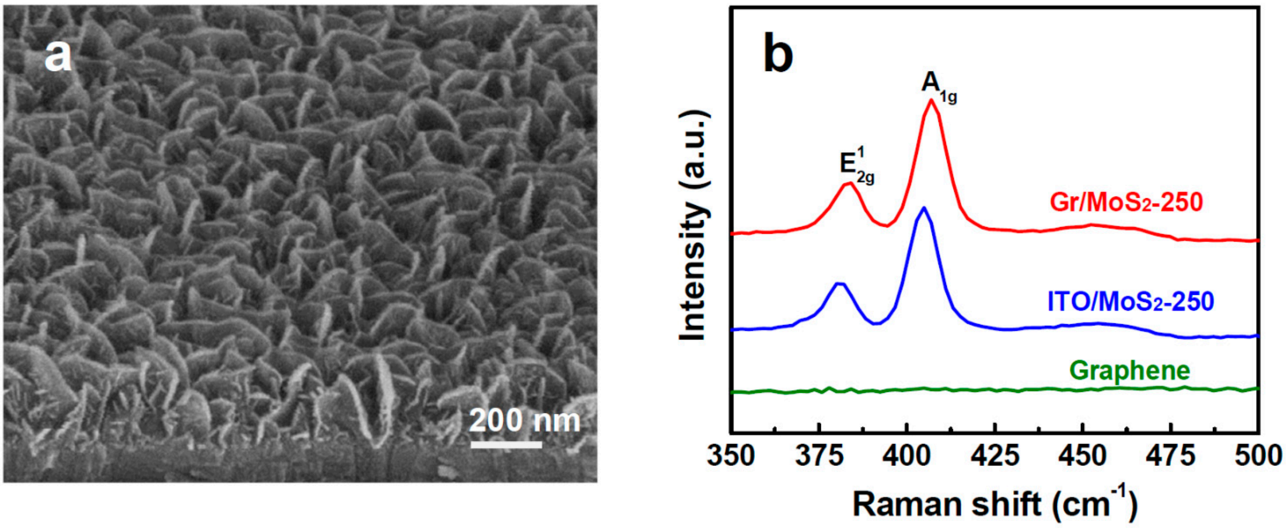


**Figure S2**. **(a)** SEM image of few-layer $MoS_2$ nanosheets grown on ITO (ITO/$MoS_2$-250). **(b)** Raman spectra of ITO/$MoS_2$-250, G/$MoS_2$-250, and pristine graphene.

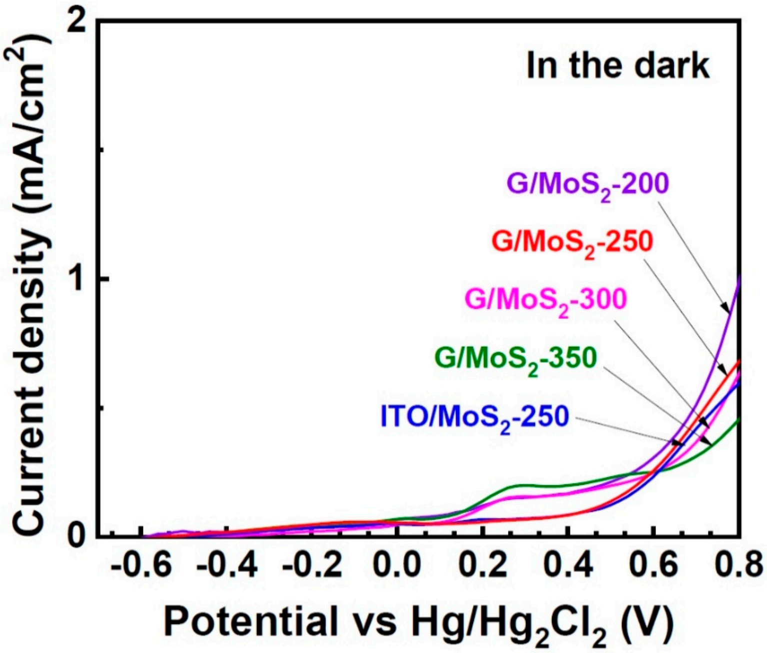


**Figure S3.** Dark current density–potential curves of PEC cells with various working electrodes (ITO/$MoS_2$-250, G/$MoS_2$-200, G/$MoS_2$-250, G/$MoS_2$-300, and G/$MoS_2$-350).
